# Information or Habit: What Health Policy Makers Should Know about the Drivers of Self-Medication among Romanians

**DOI:** 10.3390/ijerph18020689

**Published:** 2021-01-14

**Authors:** Elena Druică, Cristian Băicuș, Rodica Ianole-Călin, Ronald Fischer

**Affiliations:** 1 Centre for Applied Behavioral Economics, Department of Economic and Administrative Sciences, University of Bucharest, 030018 Bucharest, Romania; rodica.ianole@faa.unibuc.ro; 2 Department of Internal Medicine, Carol Davila University of Medicine and Pharmacy, 050474 Bucharest, Romania; cristian.baicus@umfcd.ro; 3 School of Psychology, Victoria University of Wellington, Wellington 6140, New Zealand; ronald.fischer@vuw.ac.nz; 4 Institute D’Or for Research & Teaching, Rio de Janeiro 22281-100, Brazil

**Keywords:** self-medication, cognitive determinants of self-medication, non-cognitive determinants of self-medication, knowledge, perception and practices, health policy-making

## Abstract

We use the Knowledge, Perceptions and Practices framework to analyze determinants of three types of self-medication practices in Romania: (1) self-medication in the case of cold/flu/viral infections; (2) taking non-prescribed medicine in general; and (3) self-medication based on recommendations by others. We analyzed 706 responses to an online survey and used a factor-based Partial Least Squares algorithm (PLSF) to estimate the relationships between each type of self-medication and possible predictors. Our results show that self–medication is strongly predicted by non-cognitive behavioral factors such as habits and similarity of symptoms, while cognitive determinants such as knowledge and understanding of potential risks are not significantly associated with self-medication behaviors. This paper identifies nonlinear relationships among self-medication practices and its predictors and discusses how our results can help policymakers calibrate interventions with better accuracy.

## 1. Introduction

The general attitude towards self-care in health contexts has improved over recent times [1,2,3,4,5]. Self-care practices are relevant at multiple levels [6] and include aspects such as prevention, health promotion or collaboration with health providers [7]. Within the wider sphere of self-care practice, self-medication, and specifically self-medication with nonprescription drugs, stands out as distinct through its context-dependent and often debatable nature: Is it possible to draw a line between beneficial and potential harmful practices? If yes, where and on what basis can we distinguish the positive and negative sides of the self-medication process? [8,9]. Furthermore, what are the possible factors associated with and driving self-medication practices?

From an individual perspective, the positive side of self-medication includes being the faster and cheaper, at least in the short term, treatment solutions. However, self-medication is often discussed from a healthcare reform perspective, highlighting patient independence or patient empowerment [10], which resembles related perspectives from a patient-as-consumer or pharmaceutical consumer paradigm [11,12]. Focusing on the negative aspects, most salient are issues of inaccurate self-diagnosis, incorrect dosage and use, negative interaction with other pre-existing conditions or other medications [13], the potential for pathogen resistance due to incorrect application and increased morbidity [14]. At a psychological level, self-medication can lead to choice paradoxes (e.g., over-choice) and issues around individual responsibility, which has negative consequences for wellbeing [15,16,17].

To date, there is well-developed literature around self-medication focused on low-income countries. There is less empirical evidence for middle- and high-income countries. The available evidence parallels the patterns found in low-income contexts: individuals often have a rather poor knowledge of the advantages and disadvantages of self-medication but perceive the practice positively [18,19]. While there is no widely shared theoretical framework for research into self-medication, the extant research in European countries has been concentrated around reporting the type of medicine used (the most commonly discussed are analgesics, antibiotics, digestives and brain enhancing drugs), most frequently sources of information for self-medication decisions [18] and the sociodemographic characteristics of individuals self-medicating [14,20]. One major topic of interest in recent years has been the use of antibiotics [21,22,23], especially in treating flu symptoms and colds. One common framework, as well as its variations, that has been used in the study of antibiotics use is the Knowledge–Perception–Practice (KPP) approach, which assesses what is known, believed and done about self-medication, capturing both cognitive and non-cognitive aspects relevant for self-medication behavior [24]. The main assumption underlying the model is that, by altering knowledge and perception, practices can be changed [25]. Previous studies using this framework in the area of self-medication have examined the often erroneous beliefs about the use of antibiotics [26,27], which tend to correlate with lower educational levels and appear to be more common in rural areas [28], but also pointed to a gap between knowledge and behavior in the case of healthcare (medicine and pharmacy) students [29].

There is a currently little work on possible predictors of general self-medication practices beyond research on choices for specific medications (e.g., antibiotics). Furthermore, the available research has reported differences across European countries and a relative lack of studies in Eastern European countries such as Romania [22,23,24]. Our study aimed to contribute to the literature by investigating views on self-medication practices in Romania. Romania currently ranks as an upper-middle economy, being a post-communist society with a centralized healthcare system that, despite constant reforms, has some of the worst statistics within Europe for indicators such as life expectancy and mortality rates [30]. Further complicating the situation, the low level of institutional trust (including towards hospitals and medical doctors) and an extended shadow economy [31] are likely factors that further increase the positive perceptions of self-medication as an informal beneficial practice. In line with this broader pattern, marketing studies have reported that 62% of Romanians resort to self-medication for cold or flu, headaches, joint or muscle pain, gastric burns or abdominal pain and 68% reported having an “emergency stock” of medicine to be taken in case of cold or flu, as well as pain killers and vitamins [32]. Extensive advertising, especially TV commercials, devoted to over-the-count drugs, portray self-medication as “the new normal”, enhancing perceptions of behavioral control and perceived self-efficacy and emphasizing possible benefits of the practice [33]. Most TV commercials advertising over-the-counter drugs include recommendations to carefully read the leaflet, which may warn consumers about possible adverse effects. These figures and TV commercials provide the background for recent statements by the Romanian Ministry of Health and health officials that urge Romanians to avoid self-medication, especially when the symptoms resemble those of cold, flu or infectious diseases [34]. However, are these public health communications effective? What factors are associated with self-medication in this population?

Here, we focused on three different types of self-medication: (1) self-medication in the case of cold/flu/viral infections; (2) taking non-prescribed medicine in general; and (3) self-medication based on recommendations by others. Globally, self-medication in the case of cold/flu/viral infection is the third most common form of self-medication [35]. Cold or flu symptoms often improve over relatively short periods of time even without medication, therefore, self-medication in the case of flu or cold symptoms is likely to increase the subjective efficacy of the practice, giving patients a sense of self-efficacy, which then leads to consumption of non-prescribed medication in other health contexts. Therefore, cold or flu infection may be an entry point for developing a habit of self-medication for other conditions and ailments. Furthermore, our research focused on the social aspects of self-medication. The practice of taking non-prescribed medication may be motivated through recommendations by others within one’s social network, especially in an environment where trust in official institutions is low and people tend to rely on close social networks to support each other in the absence of an efficient institutional system. To date, research has not explicitly focused on these social determinants of self-medication, which may be of importance in contexts where official health systems are perceived as inadequate.

We build on the KPP theoretical framework to explore cognitive and non-cognitive determinants of these behaviors, thus expanding previous literature that relied mainly on the role of socio-demographic determinants of self-medication [36]. Contrasting and comparing the responses to these three different questions about self-medication can also provide greater nuances and insights into the practices of self-medication. A second contribution to the literature is the consideration of possible nonlinear associations between determinants of self-medication. Previous research and health policy have generally assumed linear relationships, leading to a more is better or more effective perspective. We tested whether there are possible saturation or turning points beyond which predictors may become less relevant or important. Any such patterns should be of interest to policy makers. The rest of the paper is organized as follows. Section 2 presents the data, the measurement and the method. Section 3 presents the results. Section 4 discusses the findings and limitations.

## 2. Materials and Methods

### 2.1. Data

We collected data via an online questionnaire disseminated through Facebook, LinkedIn, WhatsApp and email in 2019. We used a combination of convenience sampling [37,38] and snowball sampling [39,40,41]. Several recent studies have demonstrated that, as the number of respondents increases, this sampling method approaches the same equilibrium regardless of the initial sampling seeds [40].

The Ethical Committee of the University of Bucharest approved the research (decision No. 14/25 February 2020). The questionnaire opens with the following information: “The participation in the study is voluntary and anonymous, and that by completing the questionnaire you provide implicit consent to participation in this research”.

### 2.2. Measurement

We focused on three types of behavior as dependent variables: (1) self-medication in the case of cold/flu/viral infections; (2) taking non-prescribed medicine in general (unspecified contexts); and (3) self-medication based on recommendations by others. The specific wordings were: “When you get a flu, you take the medicine that you have at home”; “When you get a flu, you decide yourself what medicine to buy as a cure”; “When you get a flu, you take the same medicine that you took in the past”; “You buy medicine based on advice from relatives, neighbors, friends or others”; and “Usually, you take medication that is not prescribed by a doctor”.

Concerning the possible determinants, we measured reasons and attitudes towards self-medication in the case of flu/cold/viral infections using an adaptation of a previously validated KPP questionnaire [42]. This theoretical framework captures a mixture of cognitive and non-cognitive determinants of self-medication. Table 1 shows the items and latent constructs along with their abbreviations. In addition, we measured the extent to which the respondents rely on advice from family, Internet and friends. Specifically, we asked the respondents to what extent they find the following sources reliable: (1) “My family can give me proper advice when it comes to my health issues, and they recommend proper treatment when necessary”; (2) “I can identify the right treatment for a health issue by searching for information on the internet”; and (3) “My friends can give me a proper advice when it comes to a health issue”. We measured all responses on a seven-point Likert scale, with 1 = complete disagreement and 7 = complete agreement. We also included questions on gender, education, income, medical education and age.

### 2.3. Method

First, we conducted an exploratory factor analysis to identify latent constructs within each KPP dimension (see the first three columns of Table 1). Table 1 reports the amount of variance extracted, as well as the internal consistency. One factor (Knowledge) had a Cronbach’s alpha below the recommended threshold and the average extracted variance was below recommended levels for two dimensions (Knowledge and Practice). Considering that this is an exploratory study and the first study in Romania, these values can be accepted [43]. The three items measuring the extent to which the respondents rely on family, Internet and friends in taking medicines loaded on a single factor that explained 64.3% of the variance in data and the corresponding Cronbach’s alpha was 0.84.

Given the lack of normality of our variables, we used a factor-based Partial Least Squares algorithm (PLSF) implemented in the WarpPLS 7.0 software to estimate the relationships between each type of self-medication and their potential determinants. The PLSF algorithm combines the accuracy of covariance-based SEM algorithms [44,45,46,47], with the nonparametric characteristics of classic PLS algorithms and provides estimates of the true factors as linear combinations of indicators and measurement errors [45,46,47,48]. Our estimation is based on Factor-based PLS Type CFM1, which complies with the common factor model assumptions. The advantage of the WarpPLS software over other options that conduct similar estimations is the availability to identify potential nonlinear relationships among the variables involved in the model. An application of this method via WarpPLS can add value by identifying the best curve that fits the data.

## 3. Results

Our data comprise 706 respondents, aged 18–82 (mean age 30.86, sd = 14.25). Only 16 participants were above 65 years, which is less than 3% of the total number of respondents. Out of the total sample 70% were women, 51.5% completed higher education, 59.6% were married or in a stable relationship and 13% reported medical training.

Table 2 presents the results of three models exploring how each type of self-medication behavior relates to the demographics as well as to the KPP dimensions and the items measuring the reliability of other sources. A preliminary analysis showed that between “self-confidence in administrating drugs at home” and “habits” there is a correlation of 0.79 and also a correlation of 0.8 with “practice based on similarity”. In both cases, the analysis of the variance inflation factor indicated high values (over 4), and, as a consequence, we removed the self-confidence variable from the model.

### 3.1. Demographic Variables

According to Table 2, age is negatively correlated with both self-medication in general (β = −0.181, *p* < 0.001) and self-medication based on advice of relatives, friends or others (β = −0.071, *p* = 0.018), but unrelated with self-medication in the case of cold or flu (β = −0.041, *p* = 0.112). The results for gender are similar: although women are less inclined than men to adopt self-medication when the context is unspecified (β = 0.091, *p* = 0.004), women were more inclined to take drugs recommended by others (β = −0.063, *p* = 0.031). Individuals with higher education reported greater use of self-medication for all three outcomes (β = 0.094, *p* = 0.003 for self-medication in case of flu, β = 0.070, *p* = 0.020 for self-medication in unspecified contexts and β = 0.067, *p* = 0.023 for self-medication on advice of others). Respondents with medical education are not more likely to report self-medication in case of flu (β = 0.050, *p* = 0.072) and taking drugs recommended by others (β = 0.003, *p* = 0.464) compared to respondent with no medical education. However, medical education seems to decrease self-reported tendency to take drugs without medical prescription in general (β = −0.064, *p* = 0.030). These results support the idea that colds or flu symptoms are perceived as common conditions that increase self-medication tendencies, regardless age, gender or medical education.

The perceived reliability of other sources is highly significant for statistically predicting self-medication in general (unspecified contexts) (β = 0.196, *p* < 0.001), while in the other two cases of self-medication perceived reliability of other sources does not have any impact. The result reinforces the idea that cold and flu symptoms are seen as common conditions that can be cured via self-medication.

### 3.2. The KPP Predictors

Using the KPP framework, we had information for two types of predictors. Cognitive predictors included items on the respondents’ understanding of how drug works and the risk related to self-medication. Non-cognitive determinants included items measuring habits of self-medication and self-medication because of similar symptoms. Our results indicate that the non-cognitive determinants had a greater influence compared to the cognitive predictors.

The respondents’ understanding of how the drug works predicted only self-medication in the case of flu (β = 0.062, *p* = 0.033). The second cognitive determinant, understanding the risks of taking a drug, did not predict any of the three types of self-medication studied. In contrast, practice based on similarity is highly significant in predicting self–medication in the case of flu (β = 0.524, *p* < 0.001), self–medication in unspecified contexts (β = 0.282, *p* < 0.001) and self–medication based on others’ recommendations (β = 0.390, *p* < 0.001). Habits predict both self–medication in case of flu (β = 0.250, *p* < 0.001) and self–medication in unspecified contexts (β = 0.087, *p* = 0.005).

Table 3 presents the performance indicators for each model, along with their corresponding threshold values. The results show that all three models are reliable. The first model, explaining a specific type of behavior (a specific ailment), has the highest explanatory power (53%), while the second and the third model explain less variance (32% in case of self-medication in general and 19% in case of self-medication based on recommendations).

Table 4 presents the effect sizes of each KPP predictor using the equivalent of Cohen’s f2 within the context of PLSF, assuming linear relationships. Typically, effect sizes larger than 0.02 are considered of sufficient magnitude to justify recommendations for practical intervention [49]. Effect sizes below this threshold indicate that the relationship is not practically relevant, even though it may be statistically significant [50]. Table 4 shows that self-medication practice based on similarity is not only statistically significant in predicting each of the three types of self-medication behaviors but also of sufficient magnitude to have some practical implications. The same applies to habits (in relation to flu/cold self-medication). In contrast, information on how the drug works was the only significant predictor in one of the three models, but the effect size estimate was below the minimum level recommended for practical intervention.

**Figure 1:**
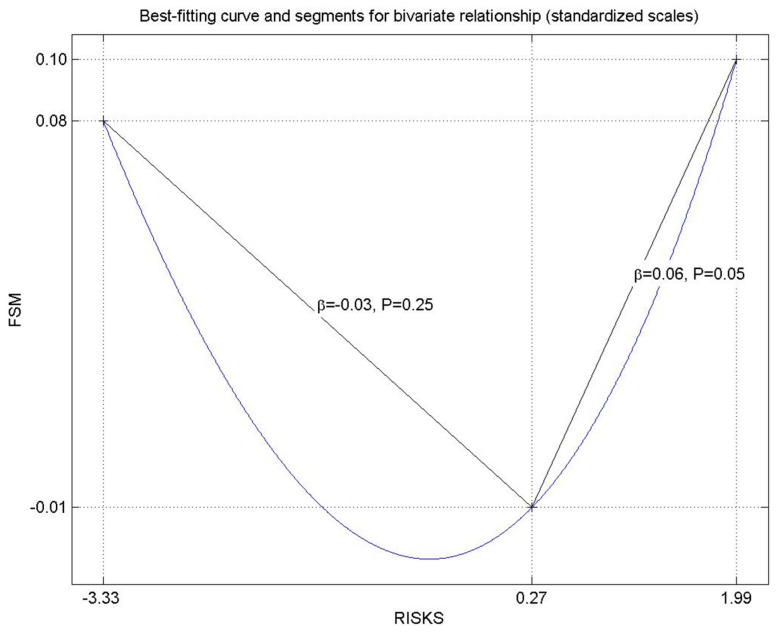
The nonlinear relationship between self-medication in case of flu (FSM) and understanding the risks and the adverse effects of the drug (RISKS).

We focused on the overall statistical patterns of our models, without including the greater complexities and insights that can be gained by using a PLSF framework. In Appendix A, we present more information on the nonlinear dynamics between our variables. To provide one example, Figure 1 shows the nonlinear relationship between self-medication for cold/flu symptoms and understanding the risks and side-effects. Overall, the linear relationship is not significant. However, as can be seen in Figure 1, the relationship follows a U-curve. Individuals below the value of 0.27 on the scaled risk latent factor do not show a reliable association with taking flu medication without prescription (*p* = 0.25), whereas those individuals with more awareness of risks show a reliable association and increased likelihood to self-medicate (*p* = 0.05). The linear effect in the whole sample was not significant.

## 4. Discussion, Conclusions and Limitations

Our paper examines predictors of three types of self-medication derived from the KPP framework. Our results show that self-medication is strongly predicted by non-cognitive behavioral factors such as pre-existing habits and similarity of symptoms. This suggests that self-medication becomes a form of routine [51]. Our findings question the use of health information campaigns as the only tool to improve or change self-mediation behavior as an important aspect of self-care health behavior. Multifaceted interventions, addressing both a wider range of determinants (e.g., behavior change interventions) and multiple target groups (including patients, medical doctors and pharmacists), should be adopted. Such multifaceted interventions have been shown to be effective in reducing antibiotics self-medication in France [52] and Poland [53] and for increasing health literacy in the wider EU [54]. According to social cognitive theory [55], knowledge of the risks and benefits of different practices is an important pre-condition in the process of building a new health practice, but additional factors are necessary to change behavior (personal beliefs, outcome expectations, facilitators etc.).

Our research found that the two cognitive determinants measured in the survey—understanding how the drug works and knowledge about potential adverse effects—did not have a significant relationship on self-medication practices after controlling for the other variables (with a single exception: a positive relationship of understanding how the drug works is observed for flu self-medication). However, we need to emphasize that this general lack of significant relationships of information related factors is not important. Certainly, information and knowledge are relevant to provide options for individuals to act upon, therefore information is important when combined with other behavioral interventions. For instance, Dusseldorp et al. [56] demonstrated that the most effective interventions in changing health behaviors are those in which the information about the behavior–health link is enhanced by prompting intention formation (defined as “encouraging the person to decide to act or to set a general goal”) and follow-up communication (defined as “contacting the person again after the main part of the intervention is complete”).

The effect sizes reported in Table 4 can provide insights for such interventions in Romania. For example, we demonstrated that the social influence of peers in shaping self-medication practices is one important driver that illustrates the limits of individual choice: individuals “are never totally independent—they are subject to a thousand influences, from those around them as well as the whole society” [57]. A wide range of studies have highlighted that the use of behavioral information (so-called nudges) focusing on social comparisons or norms is highly effective in influencing tax compliance, deterring corruption or energy saving. Such interventions are based on “eliciting social expectations with the intent of inducing desirable behavior” [58] and usually they present information on social norms. Our model focusing on self-medication as a response to social influence (advice offered by relatives, neighbors or friends) showed that social influence is salient for self-medication, yet this information is currently underexplored for self-medication interventions. There is evidence that nudging is effective for health policies [59,60]. This further highlights the importance of community-level interventions combining individual and environmental change strategies [61] for addressing behavioral health problems. For example, a school curriculum or a training program offered by patients’ organizations on health and medication literacy [62,63] could be coupled with a normative messaging intervention (describing the decisions of people from the same reference group as the target population), leveraging group dynamics in addition to individual behavior change [64].

With respect to existing habits, current literature proposes the importance to act upon the environmental factors supporting habit formation and its further reinforcement [65]. Depending on how strong the habit is, interventions could be targeted on novel or ambiguous situations when habits do not directly apply and therefore behavioral routines could be more effectively shifted. For self-medication such points may arise when people are facing a new health issue or when they change healthcare providers, e.g., family doctors, etc. A second option might be to target the larger structural context in which the habit is embedded. In the case of self-medication, this could include a closer examination of the institutional structures and services available and used by individuals within a specific context, including family medicine, health insurance, pharmaceutical services, etc. Targeting self-medication habits with a comprehensive strategy aimed at breaking habits should ideally include both options and allow for variations depending on the specific type of self-medication.

For example, self-medication for cold/flu/viral infection could be particularly effective before the beginning of the cold/flu season, by combining informational inputs with activities aimed at forming new implementation intentions (such as recommending regular annual check-ups with the family doctor and offering flu vaccination) and motivating self-control in collaboration with the medical professional. The systematic dimension may involve education campaigns (especially important for youth to develop healthy habits) and providing economic incentives (via public health insurance options or potential subsidies offered for specific patients or diseases groups). One issue to address is the relatively undefined and unregulated position of family medicine in Central and Eastern European healthcare systems, which is especially true for the case of Romania [66,67]. The gate-keeping role of family doctors is still weak, and there is a need for regaining trust for this primary healthcare link. One option to achieve this would be through improving communication and making the interaction less formal and bureaucratic. Technology can play a significant role in advancing progress in this area by facilitating the communication with family doctors via online options, formalizing digital medical prescription procedures (according to appropriate diagnosis protocols) and offering telemedicine options. The current COVID-19 pandemic has accelerated this development by increasing the presence of telemedicine and by reinforcing the role of family doctors in the larger healthcare environment [67]. In summary, the long-term goal of a health policy addressing the self-medication phenomena should be to create healthy habits around the use of medicine, which requires interventions targeting both individual and institutional factors supporting behavior change.

**Figure A1:**
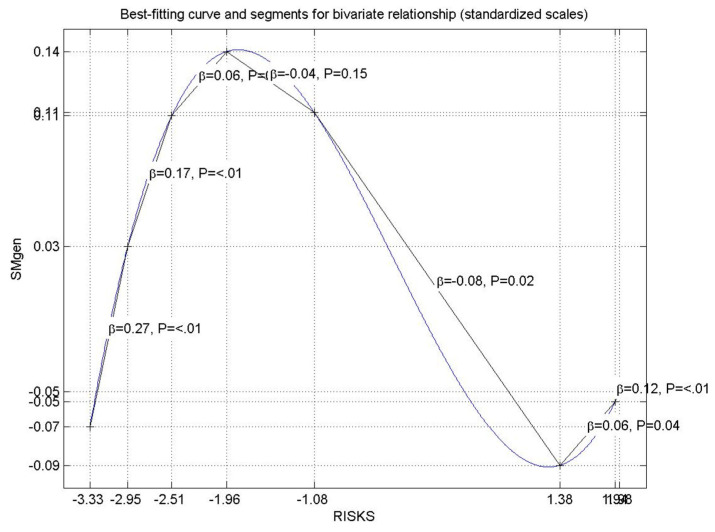
The nonlinear relationship between self-medication in general, expressed as “Usually, you take medication that is not recommended by a doctor” (SMgen), and understanding the risks and the adverse effects of the medication (RISKS).

**Figure A2:**
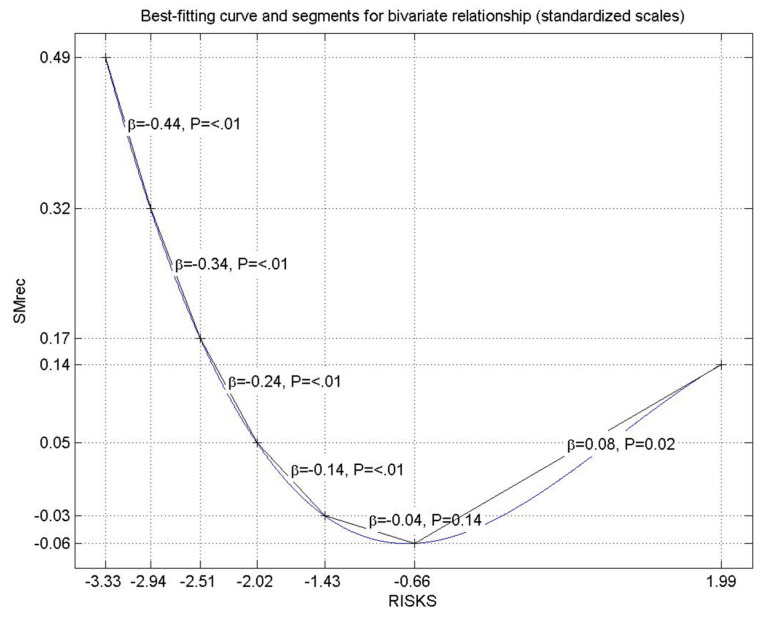
The nonlinear relationship between self-medication based on recommendations by others (SMrec) and understanding the risks and the adverse effects of the drug (RISKS).

**Figure A3:**
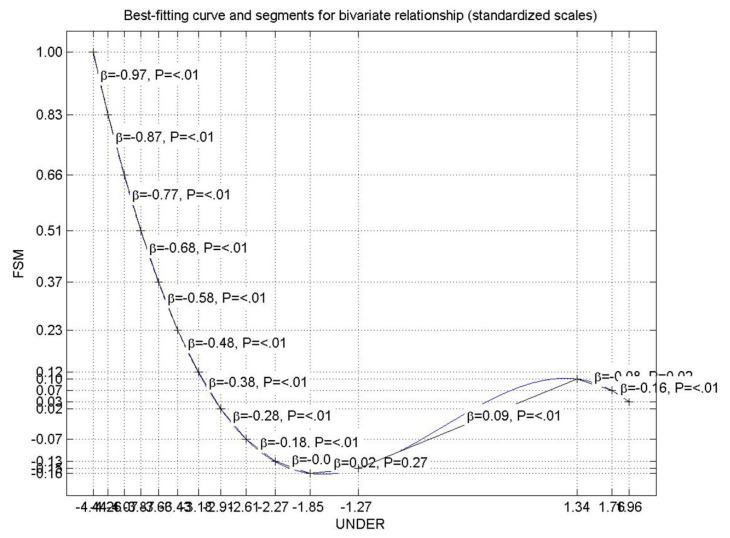
The nonlinear relationship between self-medication in the case of flu (FSM) and understanding how the drug works (UNDER).

The value of this research goes beyond the statistical significance of the predictors or their strength in supporting changes. In Appendix A (see Figure A1, Figure A2 and Figure A3), we show the lines of best fit for each model, which imply that assumptions of linearity do not necessarily hold. This is highly relevant when discussing further mechanisms why the cognitive determinants of self-medication are not significant or actionable. Figure 1 shows that understanding risks and adverse effects decreases the inclination towards self-administration of anti-flu or cold medication, but only up to a point. After that threshold, the tendency starts to increase again. This may be because the perceived knowledge gave individuals a sense of self-efficacy (being aware of the risks in general) or because the risks were judged as trivial. This in turn may have increased a sense of security in taking unprescribed medicine. The graph suggests that assumptions of a linear relationship common in traditional statistics can be misleading. The underlying pattern if confirmed in larger studies has important policy implications: most of the campaigns are meant to raise awareness about the potential adverse effects of self-medication and provide information and knowledge about possible side-effects. However, according to Figure 1, if the awareness regarding potential risks of self-medication increases, self-administration of drugs in the case of flu decreases up to a point and then increases again as individuals perceive to have more information. Hence, the paradox is that both no information on side-effects at all as well as perceptions to have adequate information can lead to increases in self-medication, challenging simple assumptions about the effectiveness of information campaigns.

The same applies to the other cases presented in Figure A1, Figure A2 and Figure A3: better understanding of how the drug works (Figure A3) or of the risks associated with taking unprescribed medication is initially associated with a decrease in self-medication practices. Figure A2 and Figure A3 show that, as the information becomes richer, self-medication practice may increase again (yet does not reach the reported levels in the absence of information). Hence, there might be an optimal level of information. Table 2 shows that only habits and practices based on similarity are relevant in predicting self-medication. If we relied on these results only, we might conclude that there is no point in providing information to people. However, the figures in Appendix A suggest that the relationship with information is more complex.

Our contribution is not without limitations. We used a convenience sample via online recruitment and the majority of our respondents were aged 18–65, with only 16 participants older than 65. Since medication might be of particular concern in older populations, our sample cannot provide much information on behavior in this important age group. In a similar vein, our sample is biased towards women (70% of the sample), and only 13% of the participants have medical education. Having collected data based on an online questionnaire, we also did not reach individuals who are less technologically savvy. Further studies need to improve this aspect by including nationally representative sampling or through targeting specific age or demographic groups.

Due to the high correlation between self confidence in administrating medication at home and patterns of habit and practice based on similarity, we decided to remove the former variable from our analysis. Future studies may explore the relationship between these variables in more detail, including an examination of possible mediation relationships between these variables. Finally, we only measured cold and flu specific self-medication responses, future studies may include other common symptoms to see whether the patterns reported here differ for other medical ailments that may be less likely to improve over time (e.g., chronic pain and headaches).

## Figures and Tables

**Table 1:** Measurement items by KPP construct.

Dimension	Items	Latent Construct	Variance Extracted	Cronbach’s Alpha
Self-medication	When you get a flu, you take the medicine that you have at home	Self-medication in case of flu	60.2%	0.82
	When you get a flu, you decide yourself what medicine to buy as a cure			
	When you get a flu, you take the same medicine that you took in the past			
Knowledge	You need to understand how a medicine works, even if it is over the counter drug.	Understanding how the drug works	43%	0.52
	The reading & understanding of leaflets in the drug pack is important before taking medicine			
	Even over the counter drugs can have adverse effects, including death	Understandings risks and adverse effects		0.74
	Our body can develop resistance against over the counter drugs			
	Continuous use of non-prescription drugs may cause dependency			
	Non-prescription drugs, most of the time, end up complicating the sickness.			
Perception	It is important to take a medicine at home as soon as I become sick	Habits	54.5%	0.8
	Taking a medicine at home is a good practice for preventing development of disease.			
	I always take a medicine as soon as I fall a sick without delay.			
	I can treat myself at home by buying medicine from the shop	Self-confidence in administrating drugs at home		0.76
	When I fall sick, there is nothing wrong with using left over medicines to treat myself.			
	Taking medication at home is an important step for keeping healthy			
Practice	I use self medication whenever I can diagnose/treat symptoms	Practice based on similarity	46.4%	0.71
	When the condition is similar to a previous sickness, I can use nonprescription drugs			
	I am always willing to use nonprescription drugs when someone whom I trust recommends them.			

**Table 2:** Estimated coefficients of a model explaining self-medication under the assumption of linear relationships.

Model	Self-Medication in Case of Flu	Usually, You Take Drugs That Are Not Recommended by a Doctor	You Buy Medicine on Advice of Relatives, Neighbors, Friends or Others
Age	−0.041 (0.112)	−0.181 *** (*p* < 0.001)	−0.071 * (0.018)
Gender Female: Reference Male	0.005 (0.445)	0.091 ** (0.004)	−0.063 * (0.031)
Education Middle: Reference Higher	0.094 ** (0.003)	0.070 * (0.020)	0.067 * (0.023)
Medical education No: Reference Yes	0.050 † (0.072)	−0.064 * (0.030)	0.003 (0.464)
Understanding how the drug works	0.062 * (0.033)	−0.025 (0.230)	−0.041 (0.115)
Understandings risks and adverse effects	0.004 (0.452)	−0.008 (0.404)	0.043 (0.101)
Habits	0.250 *** (*p* < 0.001)	0.087 ** (0.005)	0.053 (0.058)
Practice based on similarity	0.524 *** (*p* < 0.001)	0.282 *** (*p* < 0.001)	0.390 *** (*p* < 0.001)
Reliability of other sources	0.008 (0.409)	0.196 *** (*p* < 0.001)	0.003 (0.470)

*p*-Values in parentheses: † *p* < 0.10, * *p* < 0.05, ** *p* < 0.01, *** *p* < 0.001.

**Table 3:** Performance indicators for each model.

Model	Self-Medication in Case of Flu	Usually, You Take Drugs That Are Not Recommended by a Doctor	You Buy Medicine on Advice by Relatives, Neighbors, Friends or Others
Standardized mean absolute residual (SMAR) (recommended value < 0.1)	0.078	0.074	0.072
Standardized mean squared residual (SRMR) (recommended value < 0.1)	0.061	0.056	0.055
Standardized chi–squared (SChS)	0.160 (*p* < 0.001)	1.794 (*p* < 0.001)	0.767 (*p* < 0.001)
Standardized threshold difference count ratio (STDCR) (recommended value > 0.7; ideally ≤ 1)	0.995	0.994	0.994
Standardized threshold difference sum ratio (STDSR) (recommended value > 0.7; ideally ≤ 1)	0.982	0.976	0.975
R^2^/Adjusted R^2^	53%/52.5%	32%/30.8%	20.1%/19.3%
Tenehaus GoF (small ≥ 0.1, medium ≥ 0.25, large ≥ 0.36)	0.623	0.492	0.377

**Table 4:** Effect sizes of the actionable predictors of self-medication.

Model	Self-Medication in Case of Flu	Usually, You Take Drugs That Are Not Recommended by a Doctor	You Buy Medicine on Advice of Relatives, Neighbors, Friends or Others
Understanding how the drug works	0.006	0.002	0.003
Understandings risks and adverse effects	0.000	0.001	0.003
Habits	0.150	0.034	0.015
Practice based on similarity	0.361	0.140	0.163
Reliability of other sources	0.003	0.092	0.001

## Data Availability

Data is available on request.

## Appendix A

Other nonlinear relationships in predicting Self-medication.
